# Versatile and on-demand biologics co-production in yeast

**DOI:** 10.1038/s41467-017-02587-w

**Published:** 2018-01-08

**Authors:** Jicong Cao, Pablo Perez-Pinera, Ky Lowenhaupt, Ming-Ru Wu, Oliver Purcell, Cesar de la Fuente-Nunez, Timothy K. Lu

**Affiliations:** 1 Synthetic Biology Group, Department of Biological Engineering and Electrical Engineering & Computer Science, Massachusetts Institute of Technology, Cambridge, MA 02139 USA; 2 Research Laboratory of Electronics, Massachusetts Institute of Technology, Cambridge, MA 02139 USA; 3 The Broad Institute of MIT and Harvard, Cambridge, MA 02139 USA; 4 Present Address: Department of Bioengineering, University of Illinois at Urbana-Champaign, Urbana, IL 61801 USA

## Abstract

Current limitations to on-demand drug manufacturing can be addressed by technologies that streamline manufacturing processes. Combining the production of two or more drugs into a single batch could not only be useful for research, clinical studies, and urgent therapies but also effective when combination therapies are needed or where resources are scarce. Here we propose strategies to concurrently produce multiple biologics from yeast in single batches by multiplexing strain development, cell culture, separation, and purification. We demonstrate proof-of-concept for three biologics co-production strategies: (i) inducible expression of multiple biologics and control over the ratio between biologic drugs produced together; (ii) consolidated bioprocessing; and (iii) co-expression and co-purification of a mixture of two monoclonal antibodies. We then use these basic strategies to produce drug mixtures as well as to separate drugs. These strategies offer a diverse array of options for on-demand, flexible, low-cost, and decentralized biomanufacturing applications without the need for specialized equipment.

## Abstract (web summary)

The ability to combine the production of multiple biologics into a single ‘on demand’ system could help in situations where resources are limited. Here the authors demonstrate a proof-of-concept approach for the co-production of three biologics, allowing singular, mixed and combination drug products.

## Introduction

The shortage of essential drugs is of global concern^1,2^, especially in developing countries. In underdeveloped countries, governments may face budget limitations that prevent infrastructure improvement. Even in developed countries, emergency situations can compromise the supply of important medicines, such as the insulin shortage crisis in New Orleans after Hurricane Katrina^3^, or raise the risk of infectious disease outbreaks that need to be rapidly addressed. On-site, small-scale drug manufacturing can provide drugs on demand for isolated or inaccessible regions^4–6^. However, it is difficult to precisely predict the types and amounts of drugs needed in a certain region and time, so a large number of strains have to be cultivated and multiple facilities built in order to generate a large supply of needed drugs. High capital investment and maintenance costs and low utilization rates make such production difficult in regions with limited resources. Therefore, it would be of great interest to have a versatile platform to manufacture a variety of different drugs on demand and on site with low capital investment.

Biologics manufacturing involves four phases: strain/cell line construction, upstream processing (fermentation), downstream purification, and drug formulation. Usually, each biologic is produced in one strain within a manufacturing facility. Although economically efficient for large-scale production in biopharmaceutical plants, this method is inefficient and time-consuming for small-scale production, which would be useful for single-dose production, laboratory-scale research, and clinical studies^7,8^, in addition to the conditions mentioned above.

**Fig. 1:**
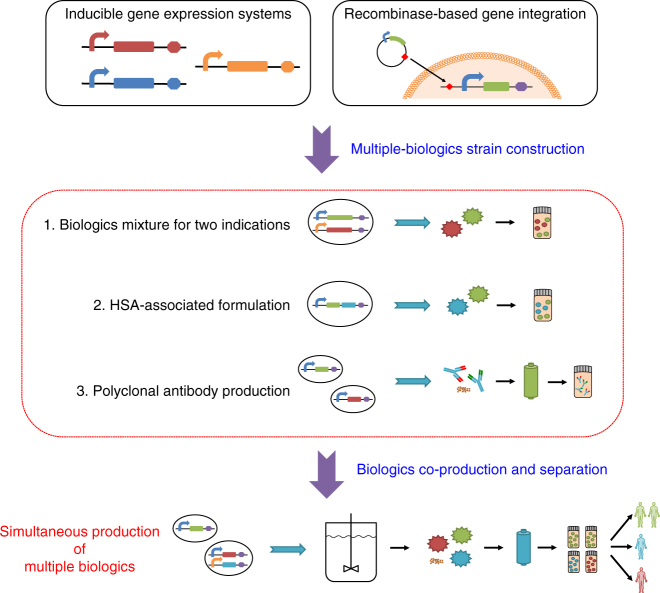
Integrated synthetic biology platform for versatile biologics production. Single-biologic or multiple-biologics *P. pastoris* strains are implemented with small-molecule-inducible gene expression cassettes integrated into the genome via recombinases. These strains produce combination drugs or multiple biologics concurrently via a consolidated, versatile bioprocessing platform

We envision that performing multiple bioprocesses simultaneously can overcome challenges in portable and/or small-scale biologics manufacturing. Here we sought to co-produce multiple drugs in a single batch via a versatile platform (Fig. 1) that: (i) generates several drugs on demand rather than one by one; (ii) enables control over the ratio of co-produced drugs and reduces the overall manufacturing time; and (iii) separates and purifies drugs in a two-stage downstream process to efficiently recover products and eliminate cross-contamination. This co-production strategy can also be used to manufacture combination drugs, i.e., drugs containing two or more active pharmaceutical ingredients. Combination drugs can have synergistic effects on a single disease or confer broad protection^9^. For example, cocktails consisting of multiple antiretroviral drugs are widely used against HIV^10^, and combination vaccines allow for fewer administrations but broad-spectrum protection against several pathogens^11^. Another class of combination drugs consists of polyclonal antibodies, which are mixtures of synergistic monoclonal antibodies (mAbs) that simultaneously interact with multiple epitopes either on the same target or on distinct targets^12–15^. For example, ZMapp, an anti-Ebola virus drug, combines three mAbs^16^; another example is the combination of lumiliximab and rituximab, which has shown enhanced antitumor effects in clinical studies^17^. Although mAb mixtures have certain advantages, such as synergistic effects and broad-spectrum protection^18–21^, the cost to manufacture them using conventional strategies is much higher than that of producing single mAbs because each mAb needs its own production strain and manufacturing equipment. Thus strategies for producing multiple mAbs and other biologics in a single batch as a co-culture should have advantages.

Chinese hamster ovary (CHO) cells are often used for biologics manufacturing^22^. However, because of their slow growth rate, CHO cells are not amenable to on-site, rapid drug manufacturing. *Pichia pastoris* is also used as a heterologous protein expression host because it: (i) can secrete large amounts of recombinant proteins using the alpha mating factor secretion signal but secretes few host proteins; (ii) grows rapidly in inexpensive media; (iii) has a eukaryotic posttranslational modification system; and (iv) is not contaminated with endotoxins or viruses^23–25^. Furthermore, glycoengineered *P. pastoris* strains with humanized glycosylation pathways are able to produce recombinant proteins and antibodies with humanized glycosylation profiles^26,27^. Synthetic biology offers a variety of platforms to regulate gene expression in various organisms. For instance, Glieder et al. developed a toolbox of synthetic promoters to systematically regulate protein expression/secretion in *P. pastoris*^28–30^. Recently, our laboratory developed a recombinase-based gene integration approach enabling the efficient insertion of large DNA fragments into the *P*. *pastoris* genome and an estrogen-inducible promoter, in addition to the native methanol-inducible promoter (AOX1 promoter)^6^. These tools were used to selectively produce either of two different biologics at a time in a portable microbioreactor platform.

Here we describe a versatile and consolidated bioprocessing platform to further streamline on-demand protein drug production. To explore the manufacturing of therapeutic protein mixtures, we designed three strategies for protein co-expression in *P. pastoris*: (i) a single strain with two inducible expression systems, (ii) a single strain with one inducible and one constitutive expression system, and (iii) two strains both having the same inducible expression system. Instead of producing each biologic separately, each strategy yielded protein mixtures produced as a single batch. We also describe the separation and purification of individual therapeutic proteins from the protein mixtures. Finally, to establish the scalability of our approach, we constructed a third inducible system and showed orthogonal inducible production of three different therapeutic proteins. These advanced inducible gene expression systems and proof-of-concept applications in protein expression provide new strategies for biologics production.

## Results

### Inducible and tunable biologics co-production

**Fig. 2:**
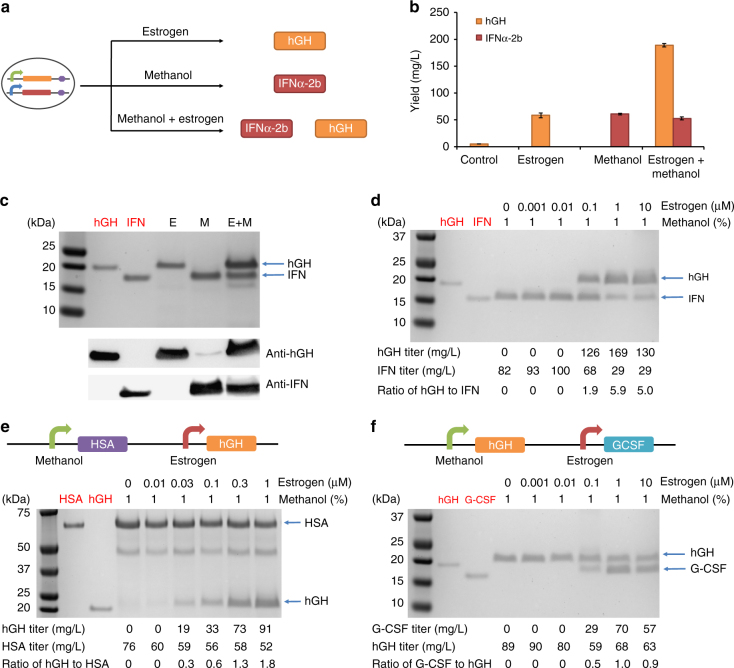
Biologics co-production with individually controllable biologic expression cassettes. E, estrogen induction; M, methanol induction; E+M, estrogen plus methanol induction. Red text above the gels indicates commercial standards while black text indicates samples obtained under induction with E and/or M. **a** Schematic illustrating the inducible production of one or two biologics from the dual-biologics production strain. **b** Titers of hGH and IFNα-2b in the supernatants of *P. pastoris* under different induction conditions. Values represent mean and s.e.m. (*n* = 3). **c** One microgram pure hGH or IFNα-2b or 30 μL supernatant of each sample was loaded in each lane and western blotting was performed with anti-hGH and anti-IFNα-2b antibodies. **d** The ratio of hGH to IFNα-2b in supernatants increased as the concentration of estrogen increased. **e** Schematic representation of the strain expressing HSA and hGH. The expression of hGH and the ratio of hGH to HSA in supernatants increased as the concentration of estrogen increased. **f** Schematic representation of the strain expressing hGH and G-CSF. The expression of G-CSF and the ratio of G-CSF to hGH in supernatants increased as the concentration of estrogen increased. The protein titers and the ratios for protein co-expression were calculated using the Image Lab software based on Coomassie blue staining

To create a flexible system to produce one or more biologics, we began by constructing a two-biologics *P. pastoris* strain (pPP363) that could be programmed to produce either human growth hormone (hGH) or interferon (IFN) alone or both proteins at once. hGH, a 22 kDa therapeutic protein used to treat growth hormone deficiency, was placed under the control of an estrogen-inducible promoter. IFNα-2b, a 19 kDa antiviral protein drug, was placed under the control of the AOX1 methanol-inducible promoter^6^. After 48 h of induction, 58 mg/L hGH was produced in the presence of estrogen, 61 mg/L of IFNα-2b in the presence of methanol, and 189 mg/L hGH and 53 mg/L IFNα-2b in the presence of both estrogen and methanol (Fig. 2a, b). The results were confirmed by Coomassie blue staining and western blotting (Fig. 2c).

Interestingly, the titer of estrogen-induced hGH significantly increased when hGH and IFNα-2b were co-expressed versus the condition in which hGH was expressed on its own. To explore this further, we tested whether the use of methanol as a carbon source could enhance the strength of the estrogen promoter or increase protein secretion. We designed three estrogen-inducible protein expression cassettes, one that expressed intracellular green fluorescent protein (GFP) (pPP255), one that secreted hGH (pPP364), and one that secreted granulocyte-colony stimulating factor (G-CSF) (pJC021). We found that estrogen-induced intracellular GFP expression was similar with or without methanol, whereas estrogen-induced secretion of hGH and G-CSF increased in the presence of methanol (Supplementary Fig. 1). The results demonstrate that methanol enhances the secretion of certain proteins in *P. pastoris*.

Having established that we could co-express two biologics in a single strain of *P. pastoris*, we then sought to fine-tune the ratio of the co-expressed proteins with our two inducible systems by varying inducer concentrations during fermentation. The two-biologics strain (pPP363) was grown for 48 h and induced with methanol and 0–10 μM estrogen. The ratio of hGH to IFNα-2b increased as the concentration of estrogen increased (Fig. 2d). To establish the generality of this observation, we constructed a strain expressing human serum albumin (HSA) upon methanol induction and hGH upon estrogen induction (pJC135) and a strain expressing hGH upon methanol induction and G-CSF upon estrogen induction (pJC034). We observed that, as the concentration of estrogen increased, the strain pJC135 produced more hGH while maintaining the same amount of HSA (Fig. 2e). This resulted in an increased ratio of hGH to HSA in the supernatant. Similar results were seen with estrogen-dependent protein co-expression with the strain pJC034 (Fig. 2f). In Fig. 2d, we observed an increase in estrogen-inducible hGH production between 0.01, 0.1, and 1 µM estrogen. This is consistent with our characterization of this system in our prior paper, which showed titrable control of reporter expression up to ~1 µM estrogen^6^. At 10 µM estrogen, we observed a decrease in hGH production; we hypothesize that this could be due to competition for resources between the two payloads at very high expression levels, which is likely to be dependent on the strain context and the specific payloads being co-expressed^31,32^. A similar effect was observed in Fig. 2f, where estrogen-induced G-CSF production also decreased at 10 µM estrogen.

It is difficult to maintain the ratio of two co-produced biologics by simply co-culturing two single-biologic strains because fluctuations during fermentation can change the growth rates of the competing strains. Researchers can perform extensive strain engineering to create microbial consortia that maintain the ratio of co-cultured strains, but it is time-consuming and laborious to optimize these systems^33,34^. In contrast, our two-biologics strategy enables dynamic control over the ratio of one biologic to another via the modulation of inducer concentrations without the need to modify strain growth rates.

### Consolidated posttranslational bioprocessing

The formulation of unstable proteins is difficult, especially for hydrophobic proteins, such as growth factors, IFNs, and cytokines^35^. To enhance solubility and reduce drug adsorption on container surfaces, excipients are used to formulate drugs. One excipient used in the pharmaceutical industry is HSA, the most abundant protein in human plasma. HSA, which can also be used as a drug, has a low risk of immunogenicity and stabilizes proteins by reducing aggregation, oxidation, and nonspecific adsorption^36–38^. However, the addition of another established cell line and manufacturing platform to produce HSA can make it costlier to produce than other small-molecule excipients (e.g., sugars, amino acids, and surfactants). Therefore, we envisioned that co-expressing a protein drug (hGH) along with HSA as an excipient in a single engineered strain of *P. pastoris* could resolve this problem.

**Fig. 3:**
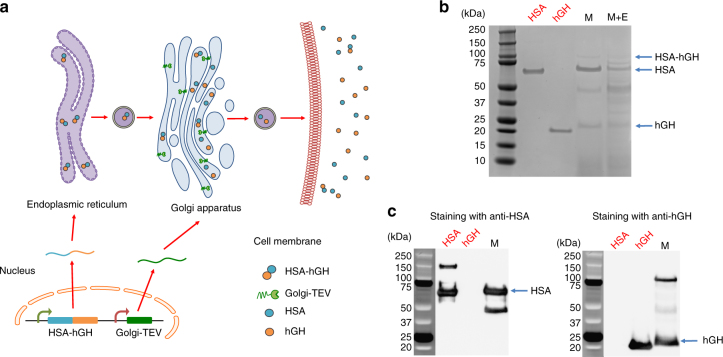
Biologics co-production with posttranslational processing. E, estrogen induction; M, methanol induction; E+M, estrogen plus methanol induction. Red text above the gels indicates commercial standards while black text indicates samples obtained under induction with E and/or M. **a** Schematic representation of posttranslational processing of HSA and hGH from HSA–hGH fusion protein. Golgi-localized TEV protease is expressed from the estrogen-inducible promoter and translocates to the inner Golgi membrane. The HSA–hGH fusion protein is expressed from the methanol-inducible promoter and enters the Golgi after synthesis in the ER. HSA–hGH is cleaved into HSA and hGH by the TEV protease in the Golgi. HSA, hGH, and a small portion of uncleaved HSA–hGH are secreted from the cell. **b** SDS-PAGE gel showing the correct processing of the fusion protein. HSA, hGH, and uncleaved HSA–hGH are labeled. **c** Western blotting with anti-HSA and anti-hGH antibodies

*P. pastoris* can effectively secrete large amounts of recombinant HSA and HSA fusion proteins^39–41^. We constructed a strain expressing two fusion proteins (pJC172): (i) HSA-hGH, consisting of an alpha-mating factor secretion signal, HSA, a tobacco etch virus (TEV) protease cleavage site, and hGH; and (ii) Golgi-TEV, consisting of a Golgi apparatus localization signal (the membrane-binding domain of alpha-1,2-mannosyltransferase) and TEV protease^26,42^. TEV protease recognizes the amino acid sequence ENLYFQ/X and cleaves between glutamine (Q) and X (P1’ site amino acid), where X can be any amino acid except proline (P)^43,44^. This feature of TEV makes it a widely used protease to produce intact proteins from fusion proteins^45^. We envisioned that the fusion protein HSA–hGH would be synthesized and folded in the endoplasmic reticulum and then would enter the Golgi before being secreted. The Golgi localization signal should direct the localization of TEV protease to the inner membrane of the Golgi, where it cleaves the ready-to-be-secreted HSA–hGH into HSA and intact hGH (Fig. 3a). Although 2A peptides have been used to secrete multiple proteins from a single cistron at the translational level^46^, our approach provides a new strategy to produce multiple biologics at the posttranslational level with only a single secretion signal.

We observed that the overexpression of intracellular TEV protease lysed the cells, so we tuned estrogen-induced TEV protease expression with estrogen and used the methanol-inducible promoter to express HSA–hGH (Fig. 3b and Supplementary Fig. 2). Our dose–response experiments revealed that basal expression of TEV protease was sufficient for effective cleavage, whereas induction of TEV expression with estrogen at a higher concentration (0.1 μM) caused cell lysis. This cell lysis could be due to the overexpression of TEV protease. Thus we induced HSA–hGH expression with methanol and allowed TEV protease to be constitutively expressed without estrogen addition. HSA–hGH was correctly cleaved by basally expressed TEV, yielding HSA and hGH, as verified by Coomassie blue staining (Fig. 3b) and western blotting (Fig. 3c). We also observed some uncleaved fusion protein, which could be explained by previous studies which showed that the processing efficiency of TEV protease is 90% when phenylalanine (F) occupies the P1’ site, since phenylalanine is the N-terminal amino acid of hGH^44^. Traditional chromatography, though not used here, could be applied to remove uncleaved fusion proteins together with host cell proteins. Our system is thus able to achieve consolidated bioprocessing of therapeutic proteins at the posttranslational level. This strategy could be potentially adapted to regulate other posttranslational processes, such as glycosylation, by replacing TEV protease with glycosyltransferases and glycan-processing enzymes.

### Single batch manufacturing of two monoclonal antibodies

**Fig. 4:**
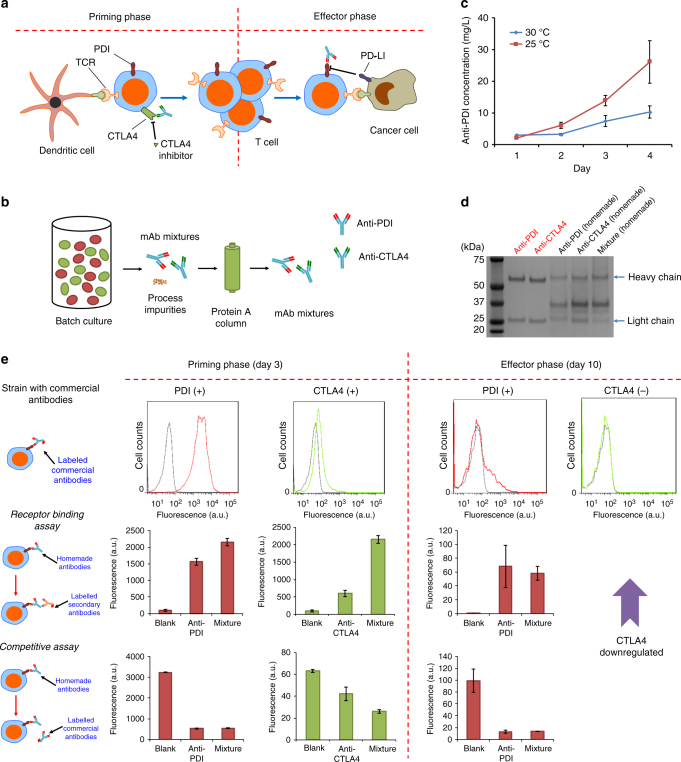
The production of mixtures of two monoclonal antibodies from *P. pastoris*. **a** Schematic representation of the effects of the two antibodies on cancer treatment. T cells activated by dendritic cells in the priming phase proliferate to enter the effector phase. The immune checkpoint inhibitor CTLA4 is expressed only in the priming phase, and the immune checkpoint inhibitor PD1 is not only upregulated in the effector phase but also present in the priming phase of memory T cells. **b** Schematic representation of the production process of the monoclonal antibody mixture. **c** The influence of culture temperature and duration on the expression of the anti-PD1 antibody. Values represent mean and s.e.m. (*n* = 2). **d** One microgram commercial anti-PD1 antibody and commercial anti-CTLA4 antibody (red text) and 10 μL of purified anti-PD1 antibody (“homemade” preparation from *P. pastoris*), anti-CTLA4 antibody (“homemade” preparation from *P. pastoris*), or a mixture of both anti-PD1 and anti-CTLA4 (“homemade” preparation from *P. pastoris*) was loaded in each lane. **e** The activities of antibody combinations were tested in cell-binding assays. Primary T cells were activated and experiments were performed after 3 and 10 days. First row: verification of the presence of the receptors using labeled commercial anti-PD1 and anti-CTLA4 antibodies. Black line: control staining. Red line: staining with commercial anti-PD1 antibody. Green line: staining with commercial anti-CTLA4 antibody. Second row: evaluation of the binding of homemade antibodies to activated primary T cells using labeled anti-human secondary antibodies. Third row: verification of the binding targets of homemade antibodies by competitive binding assays using commercial antibodies. Values represent mean and s.e.m. (*n* = 2)

Traditionally, polyclonal antibodies are made by producing each mAb separately and mixing the purified mAbs to make the final products. It was previously shown that, if conventional approaches are used, the manufacturing cost for a mixture of two antibodies is about double that for a single mAb^12,13,47^. We sought to co-culture two strains to produce antibody mixtures within a single batch in order to reduce manufacturing costs. To demonstrate a relevant proof-of-concept, we chose a mixture of two therapeutic antibodies, anti-programmed cell death 1 (anti-PD1) and anti-cytotoxic T-lymphocyte–associated antigen 4 (anti-CTLA4). Both are checkpoint inhibitor antibodies approved for treating advanced melanoma^18,21^. The targets of these antibodies, PD1 and CTLA4, respectively, both negatively regulate T cells, but they are upregulated at different stages of T-cell activation. CTLA4 is briefly upregulated in the priming phase, whereas PD1 is consistently expressed in the effector phase of T-cell activation^48,49^. The human anti-CTLA4 antibody binds to CTLA4 on the T-cell surface, blocking CTLA4 from shutting down T-cell activation in the early stage, whereas the human anti-PD1 antibody binds to PD1, preventing tumor cells from inhibiting T-cell activity (Fig. 4a). We constructed two *P. pastoris* strains that each produced one of the mAbs (pJC110 expressing anti-PD1 antibodies and pJC111 expressing anti-CTLA4 antibodies) and optimized culture conditions (temperature and time) for antibody production (Fig. 4b). We produced mixtures of these two antibodies by co-culturing the two strains. The antibodies were purified using protein G column (Supplementary Fig. 3) and then verified using sodium dodecyl sulfate-polyacrylamide gel electrophoresis (SDS-PAGE) (Fig. 4b) and western blotting (Supplementary Fig. 4).

To test the activity of these antibodies, we assayed cell surface receptor binding on human primary T cells. Human primary T cells were activated with phytohaemagglutinin (PHA) to express the cell surface receptors PD1 and CTLA4. On Day 3 and Day 10 post-induction, we analyzed the expression of the receptors using commercial anti-PD1 and anti-CTLA4. On Day 3, almost 99% of the activated T cells were expressing PD1 and 15% of them were expressing CTLA4, consistent with prior studies (Fig. 4e)^48,49^.

We then used cell-binding assays and a competitive assay to confirm the correct structures and targets of the antibodies produced in *P. pastoris*. Purified anti-PD1 antibody alone, anti-CTLA4 antibody alone, and the mixture of these co-produced two antibodies made in this study were added to the cells, and cells were then stained with labeled detection antibodies. Antibodies in all three samples bound to the activated T cells (Fig. 4e). Competitive assays with commercial antibodies binding to the two receptors were also performed to confirm that the two homemade antibodies produced in *P. pastoris* did indeed bind to their respective targets. We first incubated the cells with either homemade anti-PD1 or the mixture and then incubated the cells with phycoerythrin (PE)-labeled commercial anti-PD1. The fluorescence of the cells incubated with homemade anti-PD1 and then incubated with PE-labeled commercial anti-PD1 decreased compared to that of the cells incubated with only PE-labeled commercial anti-PD1, indicating that the homemade antibody bound to the same epitope as the commercial anti-PD1 (Fig. 4e). The same assay for our anti-CTLA4 antibody showed that this antibody bound to CTLA4 (Fig. 4e).

On Day 10, the activated T cells are expected to be in the effector phase, when CTLA4 expression is downregulated but PD1 expression is maintained. Using commercial antibodies, we observed the expression of PD1 and the disappearance of CTLA4 staining (Fig. 4e). Using homemade anti-PD1 antibodies and the antibody mixture, we then confirmed the blocking of PD1 receptors (Fig. 4e). These results indicate that the co-culture and co-purification of the antibody mixture in a single batch in *P. pastoris* could simplify the manufacturing process for antibody mixtures. Compared with mammalian hosts, the use of *P. pastoris* has the potential to decrease the time and cost needed to produce antibodies and antibody mixtures. Moreover, the ratio of two antibodies should be tunable if we were to replace the AOX1 promoter of one strain with the independently inducible estrogen promoter.

### Selective protein separation from biologics mixtures

**Fig. 5:**
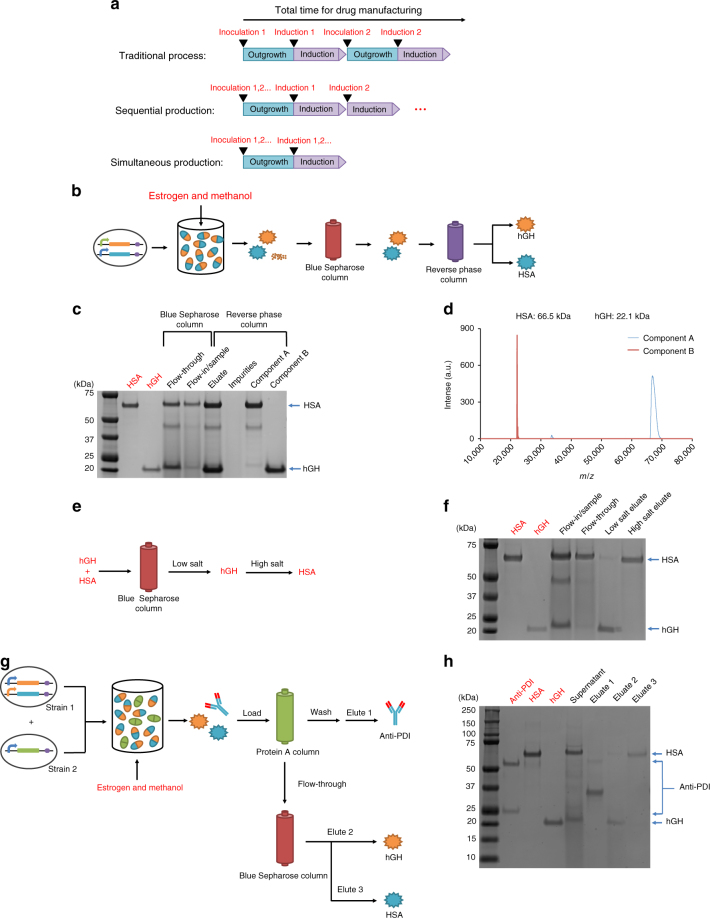
Co-production of multiple drugs by an integrated co-culture and separation process. **a** Comparison of the total time for drug manufacturing using different strategies. **b** Schematic representation of the co-production of hGH and HSA. **c** SDS-PAGE analysis of protein expression and purification. One microgram standard HSA (red text), hGH (red text), and samples (black text) were loaded in each lane. **d** MALDI analysis of HSA (component A) and hGH (component B) after purification. **e** Schematic representation of separation of HSA and hGH using Blue Sepharose column. **f** Separation of HSA and hGH from the mixed supernatant. One microgram standard HSA (red text), hGH (red text), and 30 μL samples (black text) were loaded in each lane. **g** Schematic representation of the simultaneous production of three biologics by multiplexed co-culture of a dual-biologics strain (hGH and HSA) and a single biologic strain (anti-PD1) and separation with two affinity columns. **h** The separation of the mixture of the supernatant consisting of HSA, hGH, and anti-PD1. One microgram standard anti-PD1 antibody, HSA, or hGH (red text), or 30 μL samples (black text) were loaded in each lane

Having established three effective methods to produce multiple biologics in a single batch, we sought to develop purification procedures that could be used to separate out individual therapeutic proteins from these mixtures. It is economically difficult to have multiple parallel manufacturing platforms to produce different drugs, especially in parts of the world where resources are scarce. To make multiple drugs in small quantities with only one set of manufacturing equipment, we sought to generate mixtures of biologics and then separate them through downstream processing. We expected this co-production-plus-separation methodology to take less time than existing procedures (Fig. 5a). We previously showed that we could produce two therapeutic proteins sequentially in a single manufacturing platform^6^, thus reducing the total manufacturing time from (*t*_growth_ + *t*_induction_) × 2 to (*t*_growth_ + *t*_induction_ × 2), where *t*_growth_ refers to the amount of time needed to grow the production host to high cell densities and *t*_induction_ refers to the amount of time needed to induce expression of the desired drug. Here we aimed to further reduce the time to produce *N* proteins from (*t*_growth_ + *t*_induction_ × *N*) with sequential induction to (*t*_growth_ + *t*_induction_) with simultaneous manufacturing (Fig. 5a). Downstream separation and purification should require from several hours to a couple of days for all strategies^39^. We used HSA and hGH as examples to demonstrate a prototypical workflow for the proposed simultaneous production strategy.

We first purified proteins produced by the single strain expressing HSA upon methanol induction and hGH upon estrogen induction (pJC135) with two inducible expression systems. To purify HSA and hGH in the supernatant, we used a Blue Sepharose column, which binds a variety of proteins, including albumin, IFN, lipoproteins, blood coagulation factors, and several enzymes (Fig. 5b)^39,50^. We loaded the supernatant into the column and eluted hGH and HSA with high salt buffer to get rid of most of the host cell proteins. The resulting eluate was further purified using reverse-phase chromatography, and the peaks of hGH and HSA were collected (Supplementary Fig. 5). The samples were then analyzed by using an SDS-PAGE gel and Matrix-assisted laser desorption/ionization (MALDI) (Fig. 5c, d). MALDI chromatographs indicated that the separation of hGH and HSA was virtually complete (below the detection limit). Our two-step purification strategy comprised a Blue Sepharose column (column 1) for purifying the two proteins from the host proteins and a reverse-phase column (column 2) to separate the two proteins.

To simplify purification further, the number of columns used for the separation of HSA and hGH was reduced based on the idea that proteins with different binding affinities to the Blue Sepharose column can be eluted with different elution conditions, such as salt concentration. We tested various conditions using commercial HSA and hGH samples and found that a low salt buffer (20 mM sodium phosphate and 100 mM sodium chloride) could be used to elute hGH and that a high salt buffer (20 mM sodium phosphate and 2 M sodium chloride) could be used to elute HSA (Fig. 5e and Supplementary Fig. 6). We then used the same strategy to demonstrate the separation of HSA and hGH in the supernatant (Fig. 5f). The fraction eluted first contained 92.4% hGH and 7.6% HSA, whereas the second eluate contained 95.4% HSA and 4.6% hGH, which was calculated using ImageJ. If drugs of high quality are required for further testing or clinical use, minor components and other impurities can be removed by traditional chromatographic purification processes.

To further multiplex this approach, we sought to combine multiple protein co-expression strategies. We co-cultured two strains, one strain expressing HSA upon methanol induction and hGH upon estrogen induction (pJC135) and one strain expressing anti-PD1 antibody (pJC110) upon methanol induction. Ninety-six hours post-induction, the supernatant containing HSA, hGH, and anti-PD1 was harvested and dialyzed against 20 mM sodium phosphate. We chose two commercially available columns for separation: a Protein A column was used for antibody purification, as the Fc region of antibodies binds to protein A at neutral pH and can be eluted at low pH (pH = 3.0); and a Blue Sepharose column was used to separate hGH and HSA, as described above. To separate the three proteins, the supernatant was first injected into a Protein A column. Anti-PD1 was captured in the column, whereas hGH, HSA, and the cell host proteins passed through. Anti-PD1 was then eluted by using a low pH buffer. The flow-through was then injected into the Blue Sepharose column. hGH and HSA were captured in the column, whereas the cell host proteins passed through. hGH was eluted with low salt buffer, and HSA was then eluted with high salt buffer (Fig. 5g, h). The fraction eluted first contained 86.1% hGH and 13.9% HSA, whereas the later eluate contained 89.9% HSA and 10.1% hGH, which was calculated using ImageJ. Thus we achieved primary recovery and effective separation of individual drugs from co-expressed drug mixtures, which can be followed by traditional chromatography purification processes for clinical studies.

### Orthogonal control of three biologics production

**Fig. 6:**
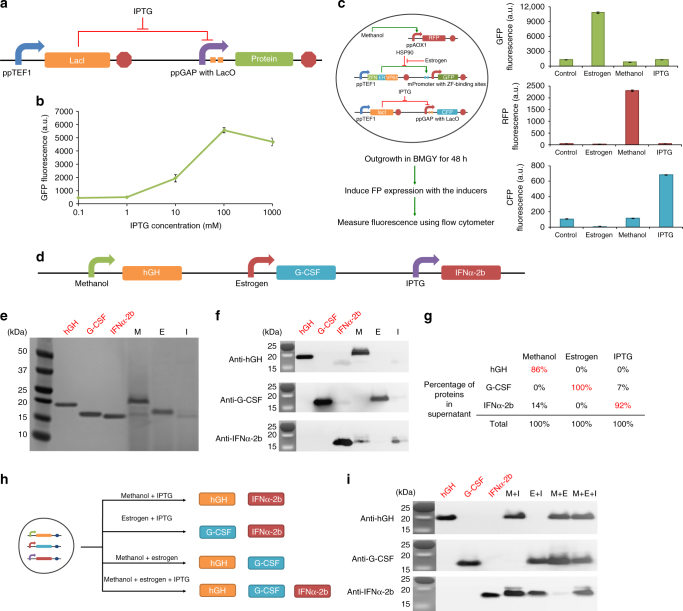
The construction of a three-biologics production strain. **a** Schematic representation of the IPTG-inducible system used in this study. This system utilizes the interaction of the *lac* repressor (lacI) and the *lac* operator (lacO). Constitutively expressed *lac* repressors bind the *lac* operator, which prevents transcription from the *P. pastoris* GAP promoter. IPTG interacts with the *lac* repressor, which releases the latter from the promoter to initiate protein expression. **b** Dose–response of GFP expression using the IPTG-inducible system. Maximum fluorescence levels were achieved with 100 mM IPTG at 48 h. Values represent mean and s.e.m. (*n* = 2). **c** The construction and testing of the strain producing three fluorescent proteins: GFP is under the control of an estrogen-inducible promoter, RFP is under the control of a methanol-inducible promoter, and CFP is under the control of an IPTG-inducible promoter. Values represent mean and s.e.m. (*n* = 3). **d** Schematic representation of inducible promoters and therapeutic proteins. **e** SDS-PAGE analysis of protein expression under different induction conditions. One microgram standard proteins (red text) or 30 μL supernatants of each sample (black text) were loaded in each lane. **f** Western blotting of the bands with antibodies for the three therapeutic proteins. One microgram standard proteins (red text) or 30 μL supernatants of each sample (black text) were loaded in each lane. **g** Analysis of the contents of each sample. Protein quantities were calculated by using ImageJ. **h** Schematic illustrating the inducible co-production of two or three biologics from the three-biologics production strain. **i** Western blotting with antibodies corresponding to the three therapeutic proteins. One microgram standard proteins (red text) or 30 μL supernatants of each sample (black text) were loaded in each lane

To demonstrate the potential scalability and generality of this approach, we designed a third gene-expression system in *P. pastoris*, which was inducible with IPTG (isopropyl β-D-1-thiogalactopyranoside). To make an IPTG-inducible promoter, we inserted a tandem repeat of two *lac* operator (lacO) sequences next to the GAP constitutive promoter. The sites of the two *lac* operators were separated by two nucleotides and placed 54 bp upstream of the start codon (39 bp upstream of the 3′ end of the promoter) (Supplementary Fig. 7). We used constitutive TEF1 to drive the expression of the *lac* repressor (LacI), which binds to the *lac* operator on the GAP promoter in the absence of IPTG, thus preventing RNA polymerase from binding and transcribing from the artificial GAP promoter. IPTG releases LacI from the promoter, initiating transcription (Fig. 6a)^51,52^. We used GFP as the reporter and constructed a *P. pastoris* strain carrying the IPTG-inducible system (pPP309). In a dose–response test, GFP fluorescence was activated six-fold in the presence of 1 mM IPTG compared to no IPTG, validating the inducibility of this system (Fig. 6b).

We then tested the orthogonality of the three systems (methanol-inducible, estrogen-inducible, IPTG-inducible) by integrating a plasmid consisting of methanol-inducible red fluorescent protein, estrogen-inducible GFP, and IPTG-inducible cyan fluorescent protein (CFP) expression cassettes into the *P. pastoris* genome (pJC101) (Fig. 6c). We induced protein expression with the respective inducers and measured fluorescence intensity by flow cytometry after 48 h. We observed the expected inducible gene expression and found that there was no cross-activation between the three inducers and the non-cognate promoters (Fig. 6c).

Having demonstrated the selectivity and orthogonality of the three inducible systems in *P. pastoris*, we sought to produce the therapeutic proteins hGH, G-CSF, and IFNα-2b. We used the methanol-inducible promoter to express hGH, the estrogen-inducible promoter to express G-CSF, and the IPTG-inducible promoter to express IFNα-2b (pJC031) (Fig. 6d). G-CSF was not stable in the medium, so we added protease inhibitors to increase its expression (Fig. 6e). The therapeutic proteins were validated and quantified by western blotting (Fig. 6f). We noted that the sizes of hGH and G-CSF appeared to be bigger than their corresponding commercial standards, which might be due to differences in glycosylation patterns. The titer of hGH was 51.2 mg L^−1^ (86% of the total therapeutic proteins) in the presence of methanol; that of G-CSF was 22.9 mg L^−1^ (100% of the total therapeutic proteins) in the presence of estrogen; and that of IFNα-2b was 9.5 mg L^−1^ (92% of the total therapeutic proteins) in the presence of IPTG (Fig. 6g). We observed IFNα-2b expression in media with methanol but did not observe CFP expression from the same IPTG promoter (Fig. 6c), consistent with the hypothesis that methanol can enhance the secretion of certain proteins but not intracellular protein expression (Supplementary Fig. 1). We also tested the co-production of biologics in the three-biologics strains with two or three inducers and observed the expected protein production corresponding to the inducer combinations (Fig. 6h, i).

## Discussion

We have developed flexible and consolidated bioprocessing schemes for integrated rapid strain engineering, inducible protein expression, and selective or combined protein purification. We showed simultaneous production of multiple biologics and combination drugs by integrating inducible protein expression systems with upstream and downstream bioprocessing in *P. pastoris*. We demonstrated inducible expression of single biologics, simultaneous production of multiple distinct biologics, co-production of protein mixtures, and ratio control for combinations. We also have presented a single-batch approach for polyclonal antibody production, which can be used for cancer immunotherapy and other therapeutic applications (Table 1). Finally, we constructed a system that allows orthogonal triple-gene control of the inducible production of three therapeutic proteins. This system can produce one, multiple, or combination proteins at a defined ratio from one strain of *P. pastoris* and with one set of production equipment in a short timeframe. The ability to produce multiple therapeutic proteins simultaneously in a single batch has the potential to significantly reduce the number of strains and facilities required for protein production, thus lowering time and expense^53,54^.

**Table 1:** Three strategies for therapeutic protein co-production

	1	2	3
Modes	Biologics co-production in single strains with individually controllable biologic expression cassettes	Biologics co-production with posttranslational processing	Biologics co-production with multiple strains
No. of strains	1	1	2
No. of promoters	2	2	1
Posttranslational processing	No	Yes	No
Case studies	Producing two distinct drugs for two indications at defined ratios (hGH and IFNα-2b)	Producing a drug of interest together with HSA, as an example of an HSA-associated formulation (HSA and hGH)	Producing monoclonal antibody mixtures for cancer immunotherapy (anti-PD1 and anti-CTLA4)

Previously, we developed a portable device to produce a single dose of two different drugs at the point-of-care, which can be used to provide medications for people in remote areas^6^. In a continuous manufacturing mode, such as perfusion culture, we can consistently produce a protein for a long period of time. Although this or other well-established on-demand strategies can manufacture a single type of drug^4–6^, additional production devices and additional cost and time are required if multiple drugs are needed for the same patient or for different patients^13^. Thus, using existing approaches, a choice has to be made between cost (using multiple devices together) and time (producing one drug at a time), both of which increase as the number of regions to be serviced and the number of people to be treated expand, because of the likelihood of concurrent needs for different drugs.

Our platform is suited not only to single drug production but also to the small-scale production of combination drugs (Fig. 4) and multiple distinct drugs (Fig. 5) at a time. Drugs can be generated as they are needed by adding the corresponding inducers during batch or continuous culture and changing the types and concentrations of inducers dynamically to meet the fluctuating demand for drugs in a certain region, for preclinical studies, or for clinical trials. Compared with the co-culture of different strains, our single-strain production strategy is able to produce one or more desired proteins in the same batch, and the ratio can be dynamically tuned by varying inducer concentrations (Fig. 2). The ability to produce mixtures of proteins could enable combination drugs or polyvalent vaccines to be made or could be used in conjunction with separation technologies to create several distinct drugs for different patients.

When multiple biologics are produced in a single facility but are not used together as combination drugs, there is the risk of cross-contamination. This risk depends on the type of the drug and can be evaluated using acceptable daily exposure (ADE) values^55^. Recently, Carver proposed a banding scheme to assess the potency or toxicity of biologics; the biologics were categorized according to their toxicity^56^. In this scheme, toxins have the lowest ADE values, and growth factors and antibodies have higher ADE values. Unlike traditional purification processes, our approach consists of two stages: separation and polishing, where one column is used to separate the proteins in the mixture. In this work, we demonstrated primary separation of antibodies, hGH, and HSA using affinity columns. These molecules can be further purified by traditional processes to remove other components, ensuring that impurities remain below their ADE levels.

One advantage of our approach compared with other small-scale or flexible manufacturing systems^4–6^ is that it can operate in existing drug manufacturing set-ups used in academia or industry. Our multiple-biologics strains can be grown in common bioreactors, and the expression of proteins of interest can be regulated by using chemical inducers. Protein mixtures can be separated and polished by adding a commercially available separation column to the purification system, which is ideally the first column to maximize recovery and purity. Protein purification systems usually consist of multiple types of chromatography and filtration, such as affinity chromatography, ion exchange chromatography, and hydrophobicity chromatography to remove impurities (mostly host cell proteins) of various characteristics and to thereby obtain high quality products. Protein mixtures can be separated using one or more columns depending on the proteins’ characteristics. Instead of developing new affinity columns or adding tags to the proteins, we can adapt common chromatography columns to purify protein mixtures of interest. In this work, we performed preliminary separation and purification for the biologics of interest and used common quality-control techniques, such as SDS-PAGE, western blotting, and cell-surface-binding assays, to assay our process outputs. For clinical studies, more comprehensive product quality-control technologies, such as peptide mapping and glycan analysis, will be important to confirm that the manufacturing processes and eventual products are consistent and of high quality.

We have constructed three orthogonal inducible systems and developed three strategies for protein co-production. Both the systems and modes can be multiplexed to meet the need for customized medications. Additional inducible systems can be designed and advanced genetic circuits can be integrated to increase the number of outputs. For example, adapting this system to utilize non-chemical inducers, such as distinct wavelengths of light, may enhance its utility. If developed as a continuous production system^6,57^, our platform should be able to produce desired proteins on demand in a dynamic fashion, reducing cost and allowing for precise control over the quantities and relative concentrations of the proteins obtained. IPTG and estrogen require freezing for long-term storage. Other inducers with greater stability characteristics could be used in the future, such as galactose and ethanol^58^. Moreover, light-inducible systems could be developed to avoid issues with inducer stability in the future^59^. Thus we envision that this platform can reduce the time and cost for producing multiple drugs and can improve access to important biologics.

## Methods

### Media and buffers

BMGY medium contained 1% yeast extract (VWR, PA), 2% peptone (VWR, PA), 100 mM potassium phosphate buffer (pH = 6.0) (VWR, PA), 4 × 10^−5^ % biotin (ThermoFisher, MA), 1.34% Yeast Nitrogen Base (Sunrise Science, PA), and 2% glycerol (VWR, PA). BMMY contained 1% yeast extract, 2% peptone, 100 mM potassium phosphate buffer (pH = 6.0), 4 × 10^−5^ % biotin, and 1% methanol (VWR, PA). YPD contained 1% yeast extract, 2% peptone, and 2% glucose (VWR, PA).

Binding buffer for Protein A, Protein G, and Blue Sepharose columns contained 20 mM sodium phosphate (pH = 7.0) (Teknova, CA). Elution buffer for Protein A and Protein G columns contained 0.1 M citric acid (pH = 3.0) (VWR, PA). Elution buffer for Blue Sepharose column contained 20 mM sodium phosphate and 100 mM sodium chloride or 2000 mM sodium chloride (VWR, PA).

### Strains and plasmid construction

The parental *P. pastoris* strain, derived from wild-type *P. pastoris* strain (ATCC 76273), was constructed before^6^. In brief, plasmid pPP074 was transformed into wild-type *P. pastoris* cells using electroporation and colonies were selected with 100 μg/mL G418 Sulfate (ThermoFisher, MA). The multiple constructs used in these experiments were built using restriction enzyme cloning and/or Gibson assembly. Plasmids are available for distribution at Addgene.

### Electroporation

Competent cells were prepared by first growing a single colony of *P. pastoris* in 5 mL YPD at 30 °C for 48 h. In all, 100 μL of the resulting culture was inoculated in 50 mL of YPD and grown at 30 °C for another 24 h. The cells were centrifuged at 1500×*g* for 5 min at 4 °C and resuspended in 50 mL of ice-cold sterile water, then centrifuged at 1500×*g* for 5 min at 4 °C and resuspended with 20 mL of ice-cold sterile water, then centrifuged at 1500×*g* for 5 min at 4 °C and resuspended in 10 mL of ice-cold 1 M sorbitol, and then centrifuged at 1500×*g* for 5 min at 4 °C and resuspended in 0.5 mL of ice-cold 1 M sorbitol (Sigma, MA). In all, 5 μg of plasmids of interest and 5 μg of Bxb1 recombinase expression vector were mixed and then added to 80 μL of competent cells and incubated for 5 min in an ice-cold 0.2 cm electroporation cuvette (Bio-Rad Laboratories, CA). Pulse parameters were 1500 V, 200 Ω, and 25 μF. Immediately after pulsing, 1 mL of ice-cold 1 M sorbitol was added to the cuvette, and the cuvette content was transferred to a sterile culture tube containing 1 mL 2× YPD. The culture tubes were grown overnight at 30 °C at 250 rpm. Samples were then spread on YPD plates (1% yeast extract, 2% peptone, 1 M sorbitol, 1% dextrose, and 2% agar) with 75 μg/mL zeocin (ThermoFisher, MA).

### SDS-PAGE and western blotting

For reducing SDS-PAGE, 30 μL of cell supernatants or purified samples were mixed with 10 μL loading dye and 4 μL 2-mercaptoethanol (ThermoFisher, MA) and heated at 90 °C for 10 min. For non-reducing SDS-PAGE, 30 μL of cell supernatants or purified samples were mixed with 10 μL loading dye and heated at 70 °C for 10 min. The samples were loaded into NuPAGE Bis-Tris pre-cast gels (ThermoFisher, MA) and run for 35 min at 200 V in MES buffer (ThermoFisher, MA).

Gels were transferred to PVDF membranes using iBlot system (ThermoFisher, MA) according to the manufacturer’s protocol. Membranes were blocked overnight using Detector Block blocking buffer (Kirkegaard & Perry Laboratories, MD) and washed three times using phosphate-buffered saline (PBS) with Tween 20 for 5 min. Membranes were incubated with primary antibodies overnight and then with secondary antibodies for 3 h. The intensity of bands was analyzed using ImageJ.

Primary antibodies used in this study: anti-hGH (ab155972, Abcam, MA): 2000X dilution; anti-IFN (ab14039, Abcam, MA): 2000X dilution; anti-G-CSF (AHC2034, ThermoFisher, MA): 2000X dilution; anti-HSA (ab84348, Abcam, MA): 2000X dilution; anti-human antibody heavy chain (MAB1302, EMD Millipore, MA): 2000X dilution; and anti-human antibody light chain (ab1050, Abcam, MA): 2000X dilution.

Secondary antibodies used in this study: Rabbit anti-Mouse IgG H&L (HRP) (ab6728, Abcam, MA): 5000X dilution; Rabbit anti-Chicken IgY H&L (HRP) (ab6753, Abcam, MA): 5000X dilution; and Goat anti-rabbit IgG (HRP) (7074 S, Cell Signaling Technology, MA): 2000X dilution.

The uncropped Commassie blue and western blotting gel images can be found in Supplementary Figures 8, 9, 10, and 11.

### LabChip protein expression analysis

*P. pastoris* cells (pPP363, pPP364, and pJC021) were inoculated (at optical density (OD) of 0.05) in 2 mL BMGY medium in 24 deep-well plates and grown at 30 °C and 800 rpm for 48 h. Cells were pelleted, resuspended in induction medium, and cultured at 30 °C at 800 rpm for another 48 h. For methanol induction, cells were supplemented every 24 h with 1% methanol. The protein titers were measured using the Protein Express Assay LabChip Kits (760499, PerkinElmer, MA) in LabChip GX II Touch system (PerkinElmer, MA) (Fig. 2b and Supplementary Fig. 1).

### Expression and purification of monoclonal antibodies

*P. pastoris* cells (pJC110 and pJC111) were inoculated into 1 mL BMGY medium and grown at 30 °C at 250 rpm overnight. The resulting culture was inoculated at OD of 0.05 into 200 mL BMGY medium and grown at 30 °C at 250 rpm for another 48 h. The cells were then induced in 200 mL BMMY medium with 1 μM pepstatin A (P5318-5MG, Sigma, MO) and chymostatin (C7268-5MG, Sigma, MO) and cultured at 25 °C and shaken at 250 rpm for 96 h and supplemented with 1% methanol and 1 μM of pepstatin A and chymostatin every 24 h. The supernatant was dialyzed in 20 mM sodium phosphate (pH = 7.0) and purified using a Protein G column (GE Healthcare, MA) according to the manufacturer’s manual. The buffer of purified antibodies was then changed to PBS (ThermoFisher, MA) using PD-10 Desalting Columns (GE Healthcare, MA) (Supplementary Fig. 4).

### Activation of human primary T cells and cell-binding assays

Human peripheral blood mononuclear cells (PBMCs) were obtained from a leukoreduction collar (Brigham and Women’s hospital Crimson Core Laboratory, MA) with gradient centrifugation. Human PBMCs were activated with PHA and cultured in Roswell Park Memorial Institute 1640 medium (ThermoFisher, MA), supplemented with 10% fetal bovine serum, 10 mM HEPES, 0.1 mM non-essential amino acids, 1 mM sodium pyruvate, 100 U/mL penicillin, 100 μg/mL streptomycin, 50 μM 2-ME, and 50 IU/mL rhIL-2 (NCI, MD) for 3 days or 10 days before being used for validating anti-CTLA4 antibody and anti-PD1 antibody production. PHA-activated PMBCs were incubated with purified anti-CTLA4 antibody and/or anti-PD1 antibody at 4 °C for 25 min, then incubated with commercial PE-labeled anti-human CD279 (PD-1) (329920, BioLegend, CA) or PE-labeled anti-human CD152 (CTLA4) (349906, BioLegend, CA). Flow cytometric analysis was done by LSRII Fortessa cytometer (BD Biosciences, CA). Data analysis was done by the FlowJo software (TreeStar Inc, OR) (Fig. 5e).

### Expression and separation of protein mixtures

*P. pastoris* cells (pJC135) were inoculated into 1 mL BMGY medium and grown at 30 °C and 250 rpm overnight. The resulting culture was inoculated at an OD of 0.05 into 50 mL BMGY medium and grown at 30 °C and 250 rpm for another 48 h. The cells were then induced in 50 mL BMMY medium with 1 μM estrogen (E4389-100MG, Sigma, MO) and 1% L81 (435430-250ML, Sigma, MO) at 30 °C and 250 rpm for 48 h, and supplemented with 1% methanol every 24 h. The supernatant was dialyzed in 20 mM sodium phosphate (pH = 7.0). In all, 5 mL of the resulting supernatant was injected into a 1 mL Blue Sepharose column and eluted using 5 mL elution buffer (20 mM sodium phosphate and 2000 mM sodium chloride, pH = 7.0). The eluted component was then concentrated using an Amicon ultra-15 centrifugal filter (UFC901024, EMD Millipore, MA) (Fig. 5c).

HSA and hGH (A7736-1G, Sigma, MO) were separated and collected using RP-HPLC under the following conditions. Column: C4; Buffer A: 0.05% TFA; Buffer B: 0.043% TFA, 80% CAN; Gradient: 5%B @5 min–100%B @45 min; Inject amount: 50 μL; Flow rate: 0.3 mL/min; Detectors: 210 nm, 280 nm (Fig. 5c).

### Protein separation using chromatographic columns

A total of 100 mg hGH and 100 mg HSA were mixed and diluted in 5 mL PBS. The solution was injected into a 1 mL Blue Sepharose column. The first fraction (mainly hGH) was eluted with 5 mL low salt buffer (20 mM sodium phosphate and 100 mM sodium chloride, pH = 7.0), and the second fraction (mainly HSA) was eluted with 5 mL high salt buffer (20 mM sodium phosphate and 2000 mM sodium chloride, pH = 7.0) (Supplementary Fig. 8). The supernatant consisting of hGH and HSA was separated as described above (Fig. 5e, f).

*P. pastoris* cells (pJC135 and pJC110) were inoculated into 1 mL BMGY medium and grown at 30 °C and 250 rpm overnight. Each of the resulting cultures was inoculated at an OD of 0.05 into 200 mL BMGY medium and grown at 30 °C and 250 rpm for another 48 h. The cells were then induced in 200 mL BMMY medium with 1% L81 (435430-250 ML, Sigma, MO) at 25 °C and 250 rpm for 48 h and supplemented with 1% methanol and with 1 μM pepstatin A and chymostatin every 24 h. The supernatant was dialyzed in 20 mM sodium phosphate (pH = 7.0). In all, 5 mL of the resulting supernatant was injected into a 1 mL Protein A Column (GE Healthcare, MA) and washed with 5 mL 20 mM sodium phosphate (pH = 7.0) and then eluted using 2 mL elution buffer (anti-PD1 antibody) (0.1 M citric acid, pH = 3.0). The flow-through was injected into a 1 mL Blue Sepharose column. The first fraction (mainly hGH) was eluted with 5 mL low salt buffer (20 mM sodium phosphate and 100 sodium chloride, pH = 7.0), and the second fraction (mainly HSA) was eluted with 5 mL high salt buffer (20 mM sodium phosphate and 2000 sodium chloride, pH = 7.0) (Fig. 5g, h).

### Flow cytometry

*P. pastoris* cells (pPP309) were inoculated at an OD of 0.05 in 1 mL of BMGY and grown at 30 °C and shaken at 250 rpm for 48 h. The resulting cultures were then cultured in induction medium with different concentration of IPTG (Gold Biotechnology, MO) for another 48 h. In all, 50 μL of the cultures was added to 500 μL PBS for flow cytometric analysis in a BD LSR II flow cytometer (Fig. 5b).

*P. pastoris* cells (pJC101) were inoculated at an OD of 0.05 in 2 mL of BMGY in 24 deep-well plates and grown at 30 °C and shaken at 800 rpm for 48 h. The resulting cultures were then cultured in induction medium consisting of methanol, estrogen, or IPTG for another 48 h. In all, 50 μL of the cultures was added to 500 μL PBS for flow cytometric analysis in a BD LSR II flow cytometer (Fig. 5c).

### 3-biologics production strain protein co-production

*P. pastoris* cells (pJC031) were inoculated at an OD of 0.05 in 2 mL of BMGY in tubes and grown at 30 °C and shaken at 250 rpm for 48 h. The resulting cultures were then cultured in induction medium consisting of methanol plus IPTG, estrogen plus IPTG (with protease inhibitors), methanol plus estrogen (with protease inhibitors), or methanol, estrogen plus IPTG for another 48 h. Protease inhibitors can increase the stability of G-CSF and was added if needed. In all, 15 μL of the cultures was loaded in each lane for western blotting experiments as described above.

### Data availability

The data that support the findings of this study are available from the corresponding author upon request. The plasmids used in this work have been deposited in Addgene (ID numbers are provided in Supplementary Table 1).

## Electronic supplementary material

Supplementary Information
