# Supplementary material for: Versatile and on-demand biologics co-production in yeast
Source: Nat Commun. 2018 Jan 8;9:77. doi: 10.1038/s41467-017-02587-w (PMC5758815; doi:10.1038/s41467-017-02587-w)
Supplement: Supplementary file 1 — Supplementary Information [file 41467_2017_2587_MOESM1_ESM.pdf]

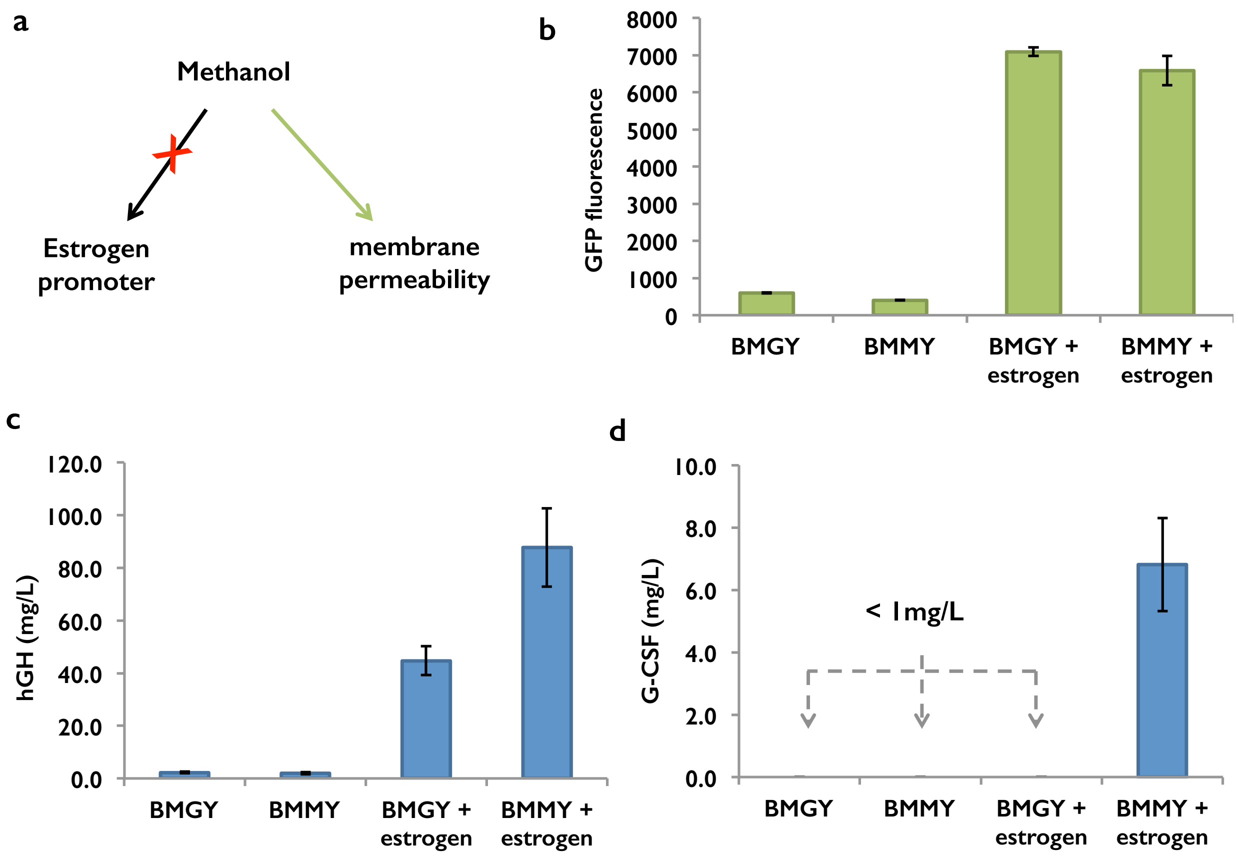

Supplementary Figure 1. The influence of methanol on estrogen-inducible protein expression. BMGY does not contain methanol, whereas BMMY contains methanol. a) Schematic illustration of the suggested mechanism. b) The addition of methanol did not increase estrogen-induced intracellular GFP expression. c) The addition of methanol increased estrogen-induced secreted hGH expression. d) The addition of methanol increased estrogen-induced secreted G-CSF expression.

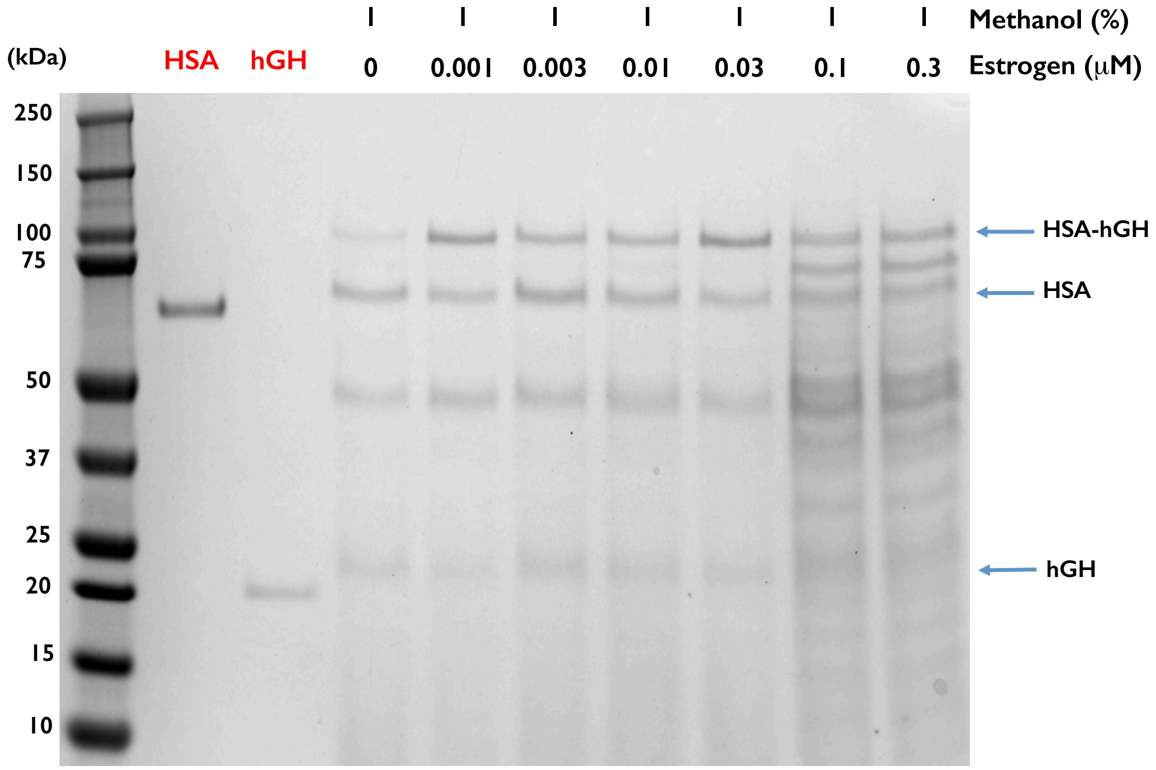

Supplementary Figure 2. **Overproduced intracellular TEV protease under the control of estrogen caused cell lysis.** The dual-biologics production strain (pJC172) was grown in BMGY for 48 hours, and then was induced with BMMY with various concentrations of estrogen for 48 hours. 1 μg HSA or hGH standards or 30 μL supernatant of each sample was loaded in each lane. The SDS-PAGE gel was stained using Coomassie Blue. Red text above the gels indicates commercial standards while black text indicates samples under the induction of methanol and various concentrations of estrogen.

a

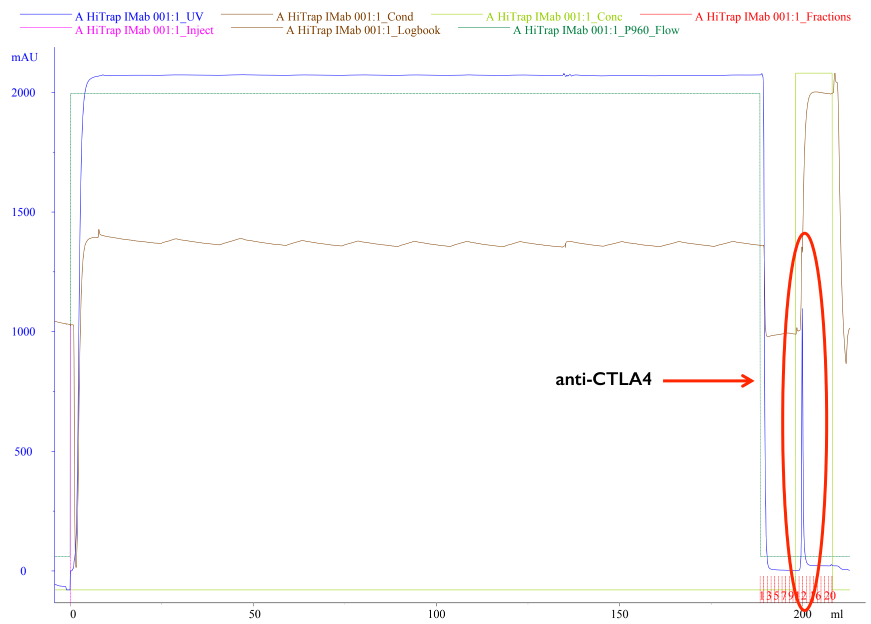

b

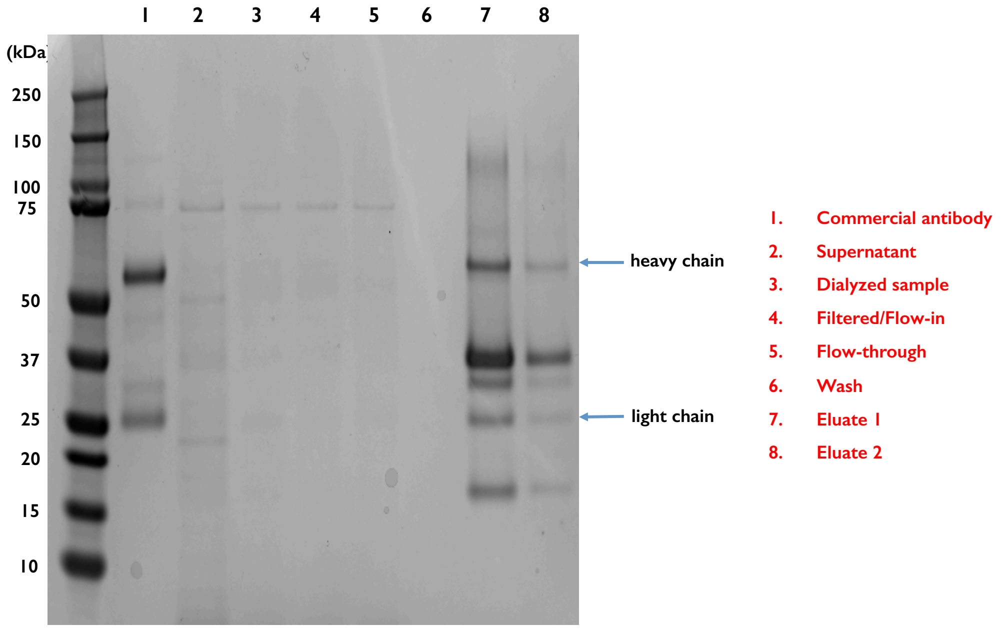

Supplementary Figure 3. **Purification of anti-CTLA4 antibodies from cell supernatant.** a) The chromatogram of the purification process using FPLC. Blue line (UV) represents the protein concentration. The peak representing anti-CTLA4 antibody is highlighted in the red circle. b) The SDS-PAGE gel of the components was stained using Coomassie Blue.

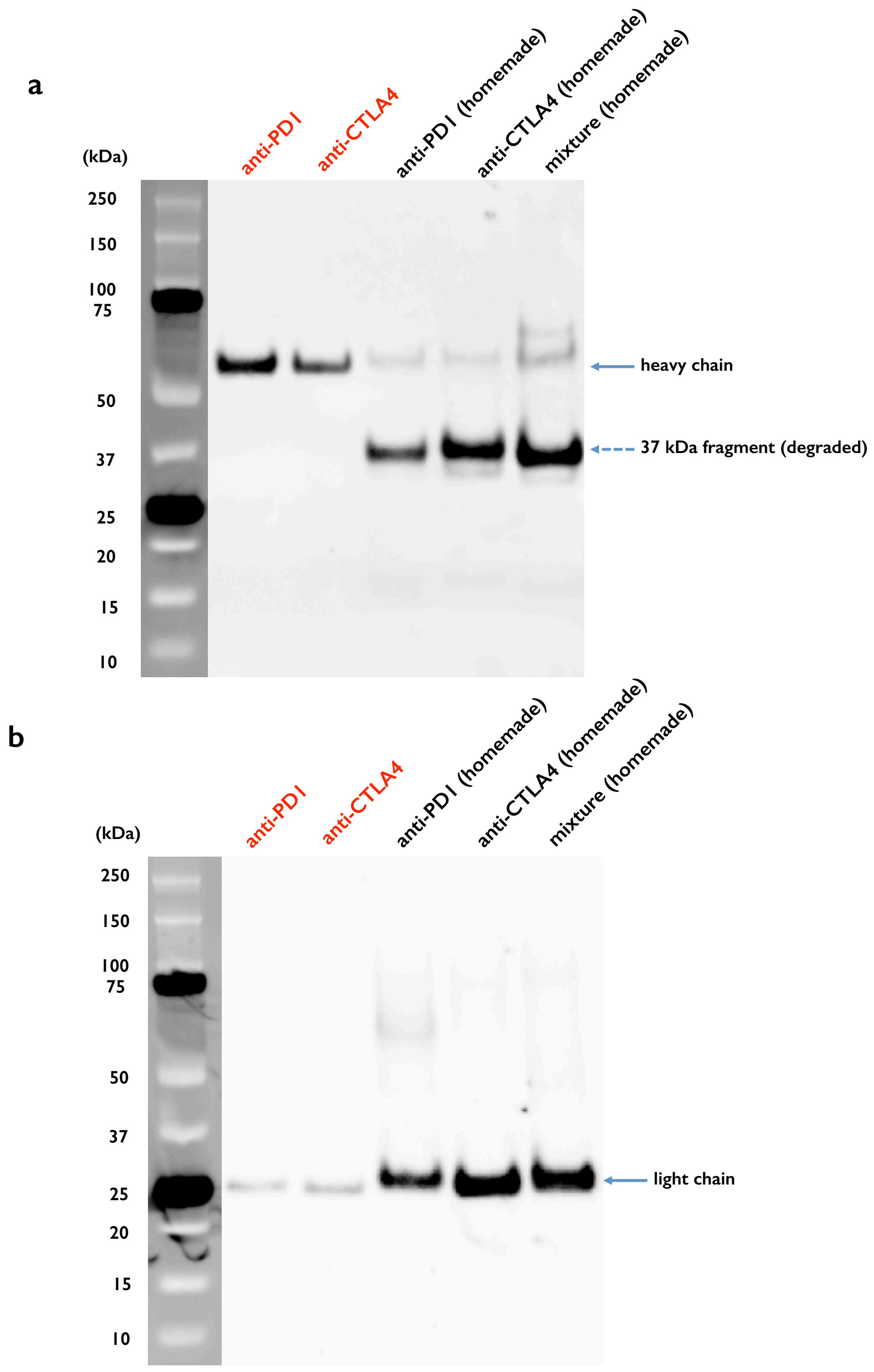

Supplementary Figure 4. **Western blotting characterization of the antibodies produced in *P. pastoris*, corresponding to 4d.** Red text above the gels indicates commercial antibodies while the black text indicates the 'homemade' antibodies that were produced in *P. pastoris.* a) Western blot of the antibodies produced in *P. pastoris* using an anti-human heavy chain primary antibody. b) Western blot of the antibodies produced in *P. pastoris* using an anti-human light chain primary antibody.

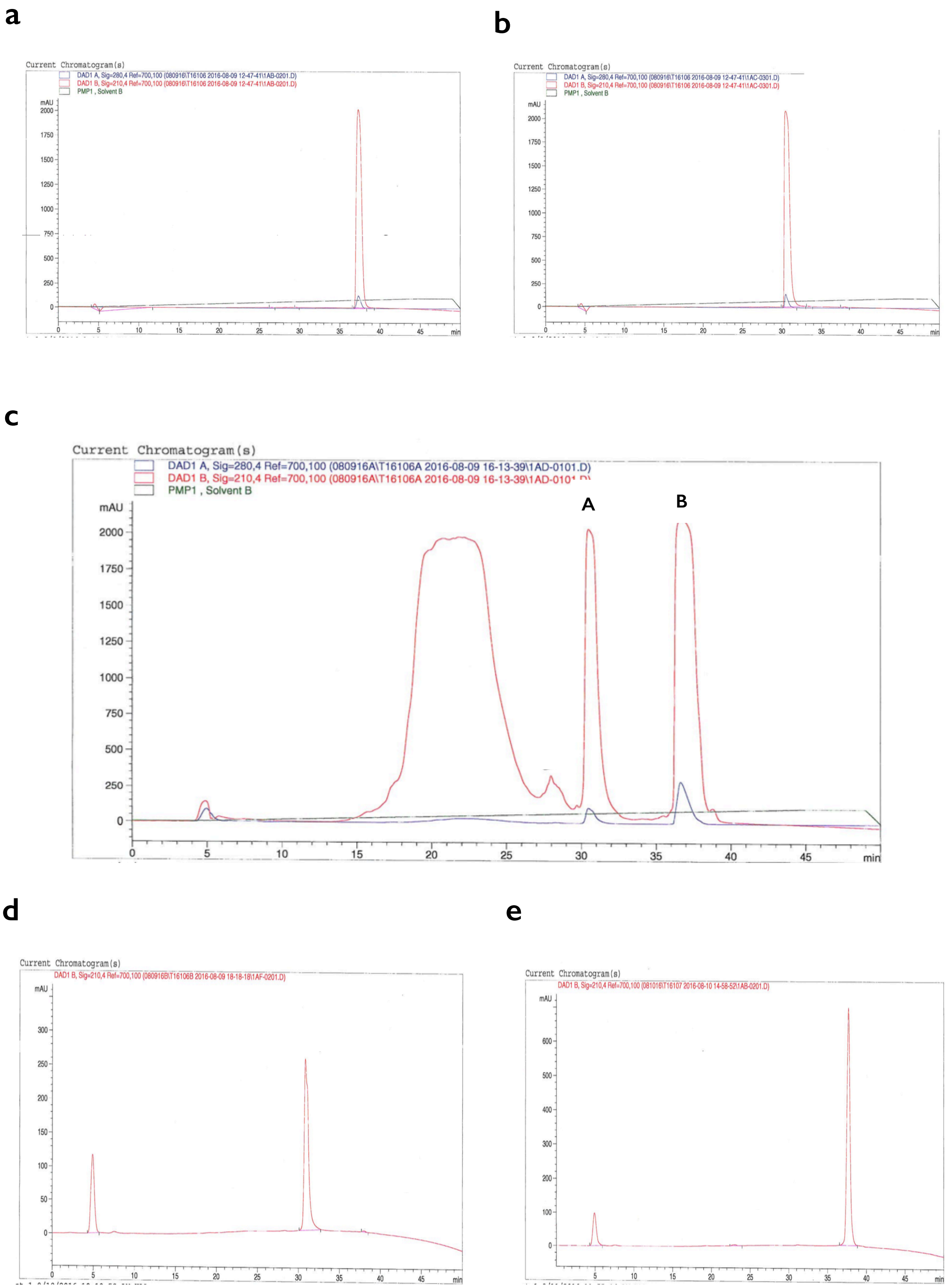

Supplementary Figure 5. **RP-HPLC purification and analysis of hGH and HSA produced in *P. pastoris*, corresponding to Figures 4c and 4d.** a) Chromatogram of commercial hGH. b) Chromatogram of commercial HSA. c) Chromatogram of the elution fraction after Sepharose Blue column purification. d) Chromatogram of fraction A from panel c). e) Chromatogram of fraction B from panel c).

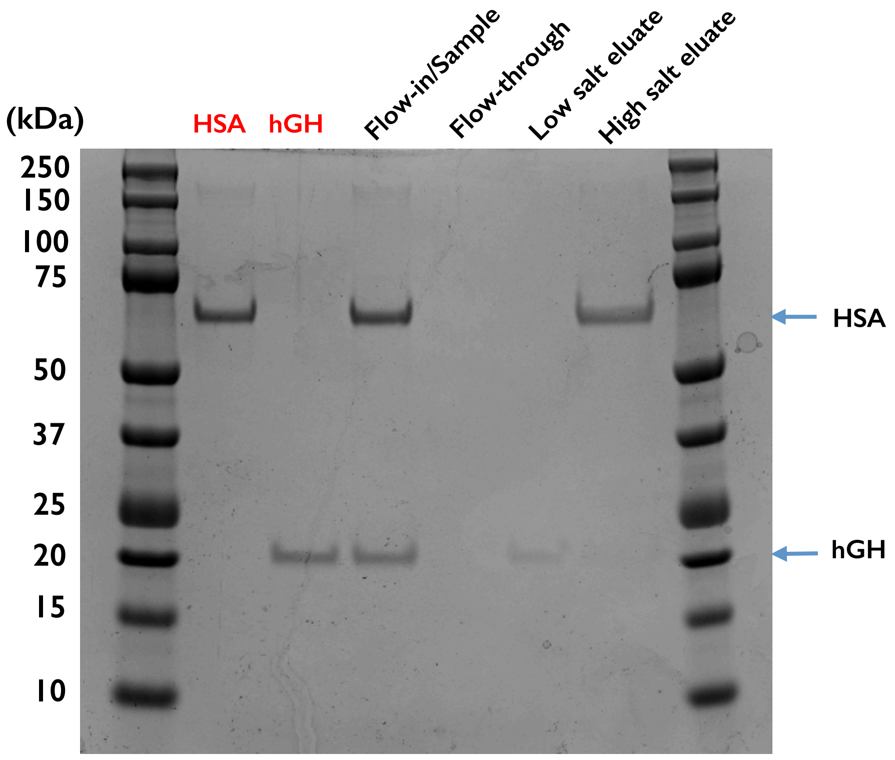

Supplementary Figure 6. SDS-PAGE analysis of the separation of the mixture of the commercial HSA and hGH using Blue Sepharose column. Red text above the gel indicates commercial standards while the black text indicates the various fractions obtained during the Blue Sepharose purification process.

CTCGAGTCAATTCTTGATTTAGTATACACATAACCAAATTTGGATCAAGTTTGAAGTA
AAACTTTAACTTCAGCTCCTTACATTTGCACTAAGATCTCTGCTACTCTGGTCCCAA
GTGAACCACCTTTTGGACCCTATTGACCGGACCTTAACTTGCCAAACCTAAACGCTT
AATGCCTCAGACGTTTTAATGCCTCTCAACACCTCCAAGGTTGCTTTCTTGAGCATG
CCTACTAGGAACTTTAACGAACTGTGGGGTTGCAGACAGTTTCAGGCGTGTCCCGA
CCAATATGGCCTACTAGACTCTCTGAAAAATCACAGTTTTCCAGTAGTTCCGATCAA
ATTACCATCGAAATGGTCCCATAAACGGACATTTGACATCCGTTCCTGAATTATAGTC
TTCCACCGTGGATCATGGTGTTCCTTTTTTTCCCAAAGAATATCAGCATCCCTTAAC
TACGTTAGGTCAGTGATGACAATGGACCAAATTGTTGCAAGGTTTTTCTTTTTCTTTC
ATCGGCACATTTCAGCCTCACATGCGACTATTATCGATCAATGAAATCCATCAAGATT
GAAATCTTAAAATTGCCCCTTTCACTTGACAGGATCCTTTTTTGTAGAAATGTCTTGG
TGTCCTCGTCCAATCAGGTAGCCATCTCTGAAATATCTGGCTCCGTTGCAACTCCG
AACGACCTGCTGGCAACGTAAAATTCTCCGGGGTAAAACTTTAATGTGGAGTAATGG
AACCAGAAACGTCTCTTCCCTTCTCTCTCCTTCCACCGCCCGTTACCGTCCCTAGG
AAATTTTACTCTGCTGGAGAGCTTCTTCTACGGCCCCCTTGCAGCAATGCTCTTCCC
AGCATTACGTTGCGGGTAAAACGGAGGTCGTGTACCCGACCTAGCAGCCCAGGGAT
GGAAAAGTCCCGGCCGTCGCTGGCAATAATAGCGGGCGGACGCATGTCATGAGATT
ATTGGAAACCACCAGAATCGAATATAAAAGGCGAACACCTTTCCCAATTTTGGTTTC
TCCTGACCCAAAGACTTTAAATTGGAATTGTGAGCGGATAACAATTTCGGAATTGTG
AGCGGATAACAATTTAATTTATTTGTCCCTATTTCAATCAATTGAACAACTATCAAAAC
ACAGAATTCATG

Features:

TEF1 promoter: 1-1136
Lac operator: 1050-1072, 1075-1097
Start codon: 1152-1154

Supplementary Figure 7. Sequence of the IPTG inducible promoter.

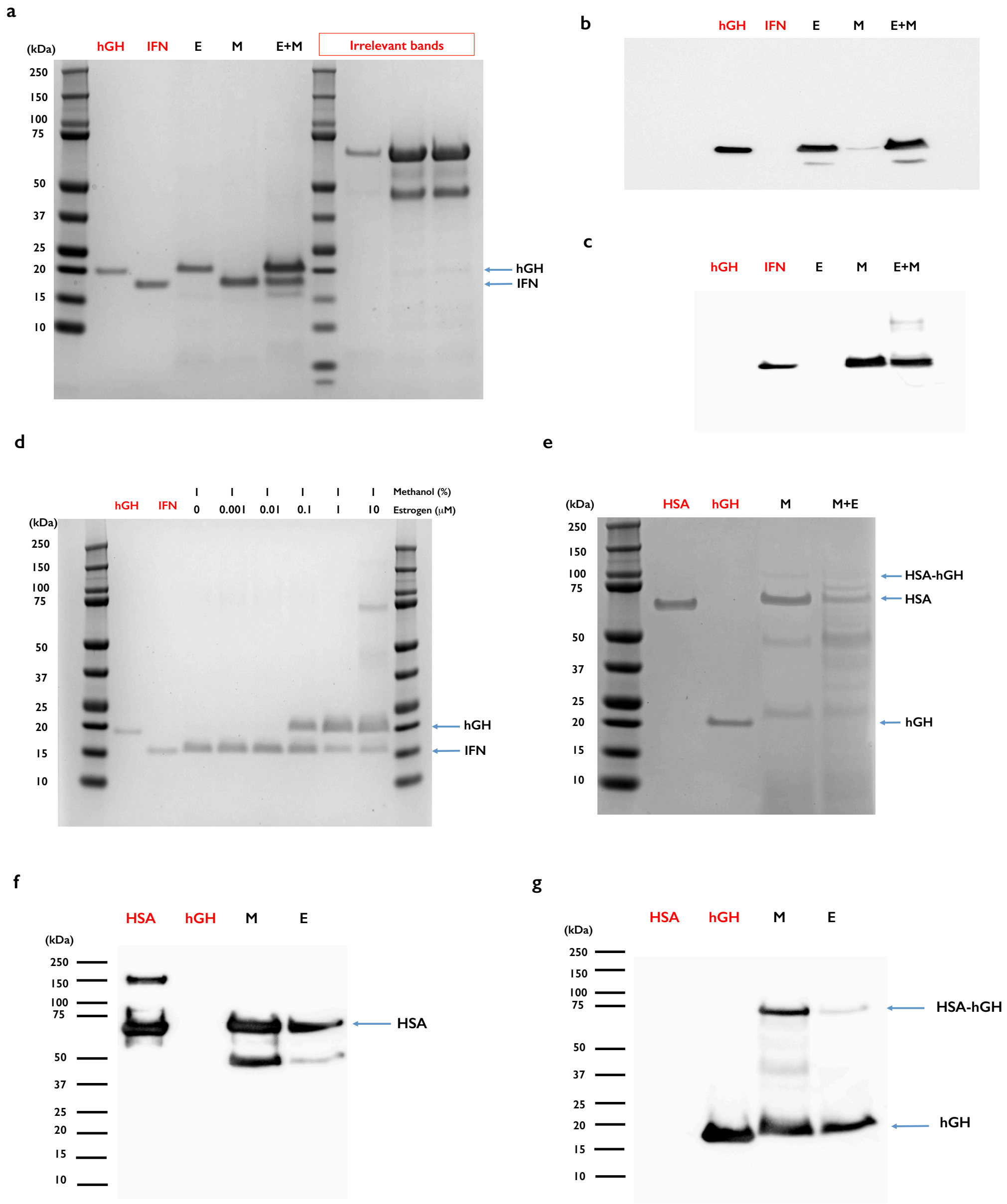

Supplementary Figure 8. **Original gel images used in Figure 2&3.** Abbreviations: E = estrogen induction, M = methanol induction, E + M = estrogen plus methanol induction. Red text above the gels indicates commercial standards while black text indicates samples under the induction of E and/or M. a) Original SDS-PAGE gel image of Figure 2c. b) Original Western blot with an anti-hGH antibody, corresponding to Figure 2c. c) Original Western blot with an anti-IFN antibody, corresponding to Figure 2c. d) Original SDS-PAGE gel image of Figure 2d. e) Original SDS-PAGE gel image of Figure 3b. f) Original Western blot with an anti-HSA antibody, corresponding to Figure 3c. g) Original Western blot with an anti-hGH antibody, corresponding to Figure 3c.

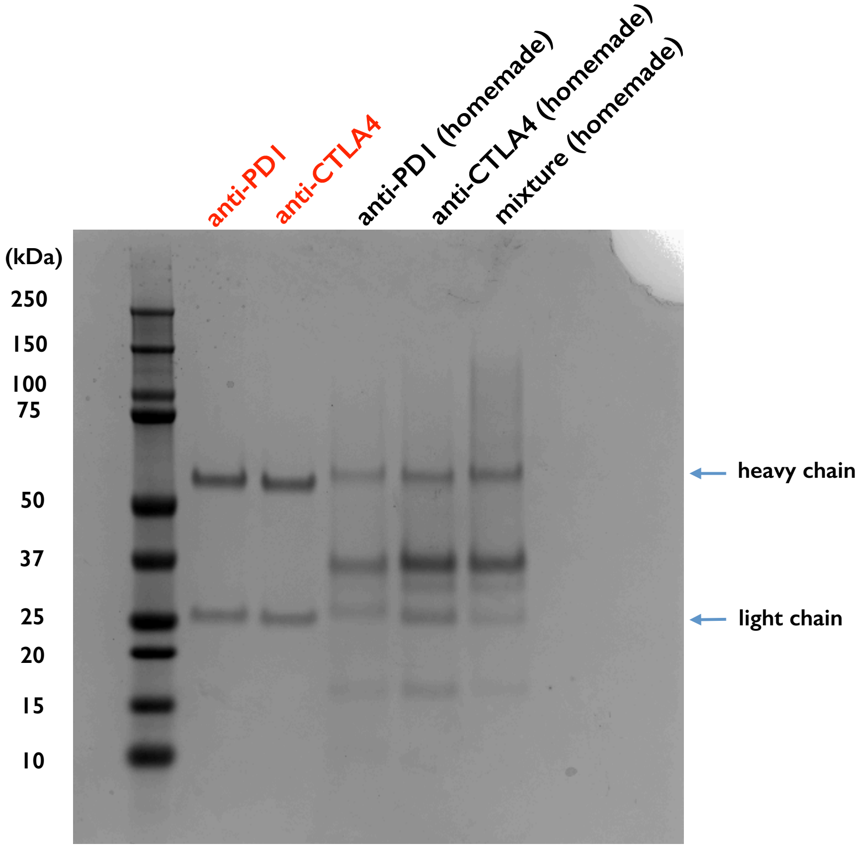

Supplementary Figure 9. Original gel image used in Figure 4d. Red text above the gels indicates commercial standards while the black text indicates homemade samples before purification.

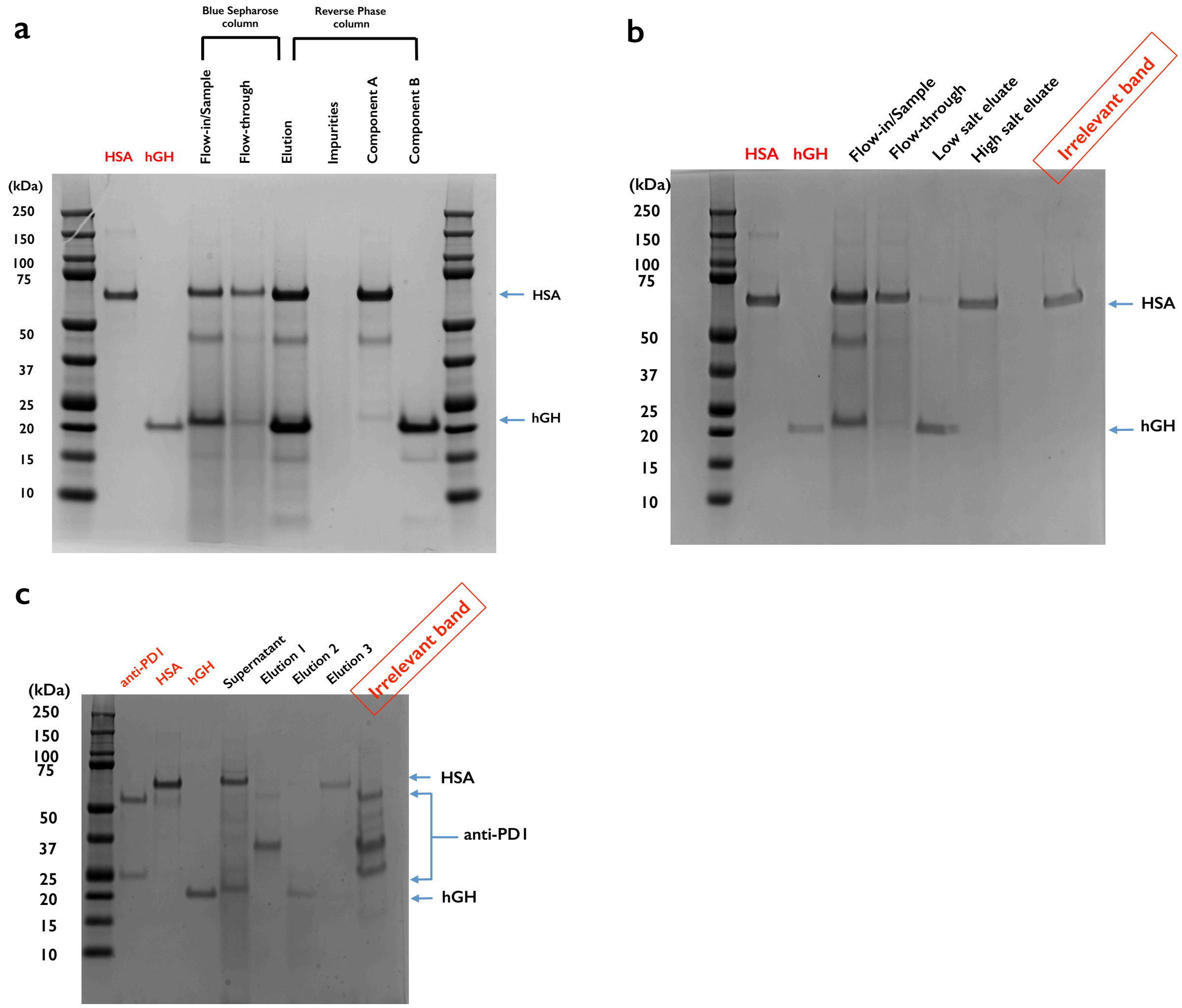

Supplementary Figure 10. **Original gel images used in Figure 5.** Red text above the gels indicates commercial standards while the black text indicates the various fractions obtained during the purification process. a) Original SDS-PAGE gel image of Figure 5c. b) Original SDS-PAGE gel image of Figure 5f. c) Original SDS-PAGE gel image of Figure 5h.

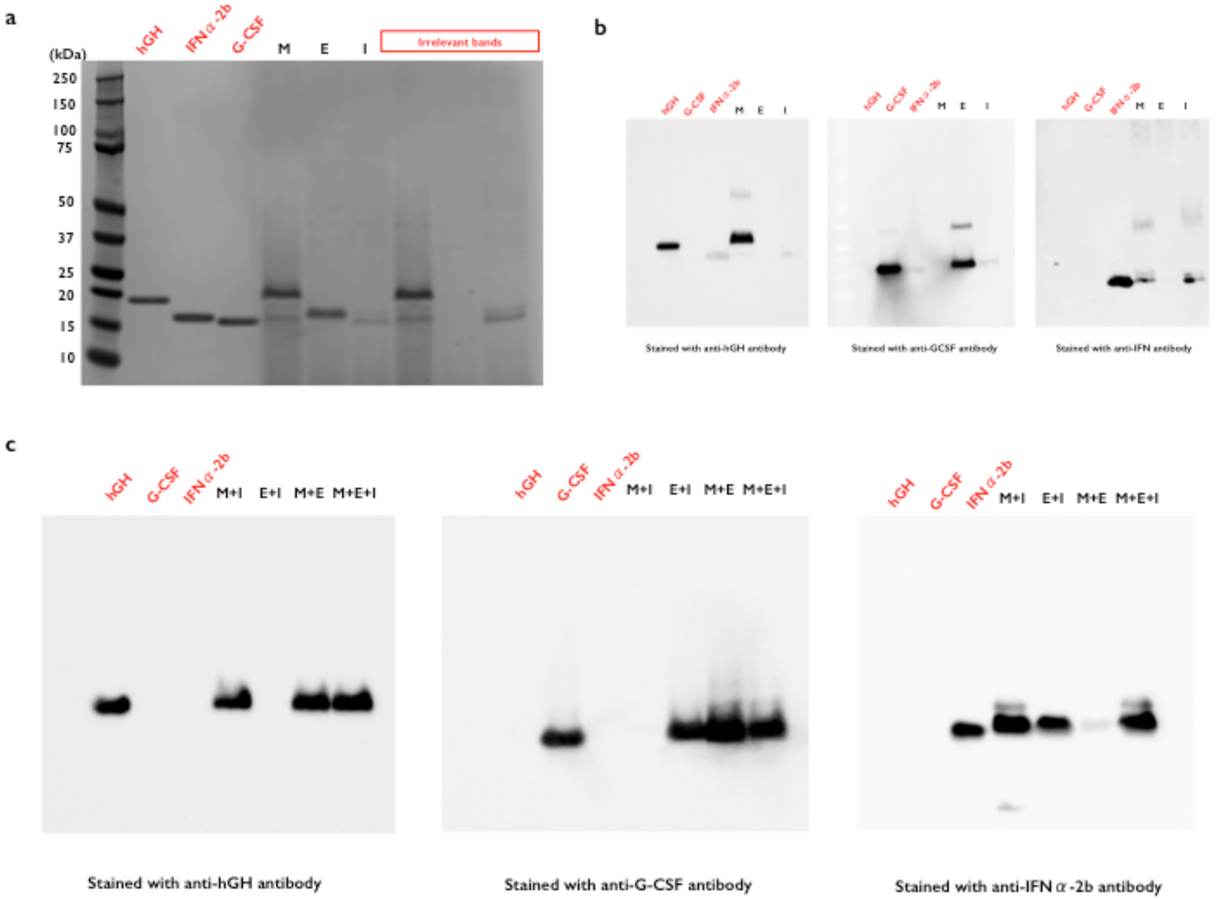

Supplementary Figure 11. Original gel images used in Figure 6. Red text above the gels indicates commercial standards while black text indicates samples under the induction of E (estrogen), M (methanol), or I (IPTG). a) Original SDS-PAGE gel image of Figure 6e. b) Original Western blots with an anti-hGH antibody, anti-GCSF antibody, or anti-IFN antibody, corresponding to Figure 6f. c) Original Western blots with an anti-hGH antibody, anti-GCSF antibody, or anti-IFN antibody, corresponding to Figure 6i.

Supplementary Table 1: List of constructs used in this study.

Figure	Plasmid Name	Addgene #
Figure 2	PP363	78984
Figure 2	JC135	104946
Figure 3	JC034	104947
Figure 4	JC172	104948
Figure 4	JC110	104949
Figure 6	JC111	104950
Figure 6	PP309	104951
Figure 6	JC101	104952
Figure 6	JC031	104953
Supplementary Figure 1	PP255	78934
Supplementary Figure 1	PP364	104945
Supplementary Figure 1	JC021	104944
